# Saturated Fatty Acids Consumed in Smoothies Increase Glucose and Metabolic Load Index in Young Adults Compared to Polyunsaturated Omega-3-Fatty Acids

**DOI:** 10.3390/nu15030642

**Published:** 2023-01-27

**Authors:** Stephanie P. Kurti, Breanna L. Wisseman, Jeremy D. Akers, Elizabeth S. Edwards

**Affiliations:** 1 Human Performance Laboratory, Department of Kinesiology, James Madison University, Harrisonburg, VA 22807, USA; 2 Human Performance Laboratory, Department of Kinesiology, East Carolina University, Greenville, NC 27858, USA; 3 Integrated Nutrition and Physiology Laboratory, James Madison University, Harrisonburg, VA 22807, USA

**Keywords:** fatty acids, postprandial lipemia, postprandial glycemia, high-fat meal, polyunsaturated fat

## Abstract

**Introduction:** Chronic diets high in saturated fat (SF) and omega-6-fatty acids (O6FAs) elevate fasting triglycerides (TRGs) and glucose (GLU). Postprandial TRGs, GLU, and Metabolic Load Index (MLI) are better predictors of disease risk compared to fasting levels alone. Conversely, diets high in omega-3 fatty acids (O3FAs) may be cardioprotective. Unfortunately, many existing postprandial studies are not standardized to body weight and given in an amount individuals would typically consume in their daily lives; the MLI is not calculated, and varying types of fat content are not examined. Therefore, we sought to determine whether SF, O3FAs, or O6FAs altered postprandial TRGs, GLU, and MLI from a standardized mixed meal. **Methods:** Fifteen individuals (6 M and 9 F) visited the laboratory three times, separated by at least 48 h, to consume HFM smoothies with varying FA composition (SF, high O6FAs, and high O3FAs). The smoothies were standardized to 12 kcal/kg body weight, 63% total fat, and 0.72 g/kg sugar. TRGs and GLU were collected at baseline and at 2 h and 4 h postprandially; the MLI was calculated by summing the TRG and GLU responses at each time point. **Results:** There was a significant increase in TRGs across time points (*p* < 0.001). For TRGs, there was a trend toward a significant interaction between smoothie type and time (*p* = 0.06) due to the increase in TRGs in the SF compared to the O3FA smoothie. There was an increase in postprandial GLU that varied across smoothie types (*p* = 0.036). Taken together, the MLI was elevated in the SF smoothie compared to the O3FAs at 2 h (*p* = 0.041). **Conclusion:** A SF smoothie in the morning elevated the metabolic load compared to an O3FA smoothie. Mechanisms of action in the competing clearance of TRGs and GLU warrant further investigation.

## 1. Introduction

The Westernized diet is characterized by poor dietary patterns, specifically consuming processed foods, saturated fat (SF), and omega-6-polyunsaturated fatty acids (O6FAs), combined with low fruit and vegetable intake [1]. High levels of dietary SF and an increased ratio of O6FAs to omega-3-polyunsaturated fatty acids (O3FAs) may be proinflammatory [2], subsequently increasing CVD risk [3,4]. Conversely, a higher O3FA to O6FA ratio may be anti-inflammatory and have a cardioprotective effect [2]. While chronic diets high in SF, O3FAs, and O6FAs may alter fasting triglycerides (TRGs) and glucose (GLU), it is well documented that how the body responds to a meal, termed the postprandial response, is a better indicator of overall disease risk compared to fasting levels alone [5,6]. Even a single HFM increases postprandial lipids, which, when consumed chronically, may elevate LDL-C particles, which can infiltrate the endothelium and initiate atherosclerotic plaque development [7]. Because many individuals consume multiple high-fat meals a day, often with snacks in between [8], they are spending most of their day in the postprandial state [9].

Many studies have shown that HFMs high in SF elevate postprandial TRGs and GLU, increasing the metabolic load experienced from the meal [10,11,12,13]. The summation of postprandial TRGs and GLU is termed the Metabolic Load Index (MLI) [13]. The MLI may be a better indicator of the entire meal response because the typical Western diet is characterized by the consumption of mixed meals high in both TRGs and GLU, which compete for clearance from the stomach, small intestine, bloodstream, and peripheral cells during the postprandial period. While our group utilized acute exercise interventions to lower adverse postprandial TRGs, GLU, and MLI [12,14], modifying the type of fatty acids consumed has shown mixed results in attenuating postprandial metabolic outcomes. No existing studies to our knowledge have looked specifically at fatty acid compositions among SF, O3FAs, and O6FAs on metabolic load, though much has been done to investigate postprandial TRGs alone. A recent study by Sciarrilo and colleagues showed that SF and monounsaturated fatty acids (MUFAs) did not have differential responses on the MLI in healthy adults [15]. These results are contradictory to studies that show lowered postprandial TRGs after meals with high MUFAs and PUFAs [16,17]. In some cases, increased postprandial TRGs have been documented in meals high in MUFAs and PUFAs compared to SF meals [18]. With these results taken together, there appears to be substantial variability in the existing literature. The present study adds to the existing data to better understand whether SF, O6FAs, and O3FAs have differential postprandial responses, specifically in examining the TRGs, GLU, and metabolic load from the test meals rather than these responses alone. In addition, many of the test meals used in the literature are not true-to-life or standardized to body weight to represent what an individual would consume in their daily life. Considering smoothies are a popular meal choice for young adults in the morning, commonly made with milk, sugar, oils, and fruit, a secondary purpose was to examine HFMs with added fruits and minimally processed ingredients relative to the subject’s body weight to explore whether a “true-to-life” HFM would alter postprandial metabolic responses. We hypothesized that the O3FA smoothies would exhibit the lowest metabolic load when compared to the O6FA and SF smoothies, though differences may not be present in TRGs or GLU alone.

## 2. Materials and Methods

Healthy, college-aged students completed four testing days, which included a familiarization session followed by three sessions, which were randomized and double-blinded and employed a cross-over design (*n* = 15, 6 M/9 F). These methods were part of a larger study investigating airway inflammation after the consumption of varying fatty acids (Wisseman et al., under review) and were approved by the Institutional Review Board at James Madison University. For ease of interpretation, tables and methods are replicated below. Inclusion criteria were between 18–35 years old; no diagnosed cardiovascular, metabolic, pulmonary, or renal disease; and not on anti-inflammatory, antioxidants, supplements, or medications that may impact blood lipids.

### 2.1. Familiarization Session

The familiarization session included informed consent, as well as completion of the International Physical Activity Questionnaire (IPAQ) to represent a typical 7 days of physical activity. The IPAQ is a questionnaire to assess chronic PA level for a “usual week” and has been validated against accelerometry data [19]. Height was measured with a stadiometer (Charder Model HM 200P, Charder Electronic Co Ltd., Taichung, Taiwan) and weight with a standard physician’s scale (Dymo Pelouze model 4040, Newell Brands, Hoboken, NJ, USA). Waist circumference was measured by a trained investigator prior to the subjects completing a dual X-ray absorptiometry (DEXA) using GE Lunar iDXA (Fairfield, CT, USA).

### 2.2. Smoothie Composition

After the initial visit, participants came into the laboratory for three high-fat meal challenges using smoothies designed and prepared by a Registered Dietitian Nutritionist at James Madison University within three days of smoothie consumption. All ingredients were measured to the nearest gram with a Detcecto digital scale (Web City, MO, USA). Smoothie composition can be found in Table 1. Each smoothie contained ~12 kcals/kilogram of body weight (0.72 g/kg body weight sugar, ~63% fat) but varied in the fruit added and fatty acid composition (i.e., strawberry—SF; blueberry—O3FA; and peach—O6FA). The SF smoothie was made with butter, whole milk, heavy cream, strawberries, and sugar. The O3FA smoothie was made with flax seed oil, 2% milk, blueberries, and sugar, and the O6FA smoothie was made with safflower oil, 2% milk, peaches, and sugar. Researchers and participants were blinded to the fatty acid composition in the smoothies until the end of the study period.

### 2.3. High-Fat Meal Challenges

Prior to each HFM, participants were required to abstain from exercise for 48 h before each of the smoothie challenges and replicate their baseline diet 24 h prior to each of the smoothie challenges. When subjects came in for their session, investigators ensured these criteria were met. Baseline blood pressure was taken after the participant was seated for 5 min, and then an initial blood draw through fingerstick procedure was performed. Blood was analyzed with a CardioCheck Plus Analyzer (PTS Diagnostics, Indianapolis, IN, USA). Each blood glucose measure required 15 microliters, while blood lipids required 40 microliters of blood sample. Participants had 20 min to consume the smoothie after the baseline measurements were taken. The time for the sessions started immediately upon completion of the smoothie. The participants were all able to complete the familiarization session and the three smoothie conditions. Finger sticks were performed to assess TRGs, GLU, total cholesterol (TC), high-density lipoprotein cholesterol (HDL-C), and low-density lipoprotein cholesterol (LDL-C) at baseline, 2 h, and 4 h after the smoothie consumption. The Metabolic Load Index was characterized by the summation of TRGs and GLU [13].

Statistical analysis was performed using GraphPad Prism. Sample size calculation was performed a priori based on our previous research using significant differences in triglycerides and airway inflammation. With a power of 0.80 and a two-sided alpha of 0.05, 12 subjects were required to complete all three conditions to observe differences in airway inflammation (data under review and not included in the present study). After collection, data were analyzed for normality, and all data were normally distributed. A three-way repeated measured analysis of variance (RM-ANOVA) was run with the time (baseline, 2 h, and 4 h) as the within-subjects effect and the fatty acid smoothie condition (SF, O3FAs, and O6FAs) as the between-subjects effect. Total AUC for TRGs, GLU, and MLI were also calculated. Tukey’s multiple comparison tests were run to assess pairwise comparisons between time points and conditions. Significance was set to *p* < 0.05 for all analyses.

## 3. Results

Participant characteristics can be seen in Table 2. Males had a significantly lower body fat percentage compared to females (18.4 ± 6.8%; females: 30.8 ± 10.3%; *p* = 0.022). Participants tended to be physically active based on their IPAQ scores, with moderate to vigorous physical activity in the ranges of 3.5–10.3 h of MVPA/week. The range in kcals participants consumed in the smoothies was 624–1238, with a mean of 890.8 ± 172.8. The range in kcals consumed in the O3FA smoothie was 187.2–371.7, in the O6FA smoothie 261.9–520.2 kcals, and in the SF smoothie 238.5–473.4 kcals.

**Figure 1:**
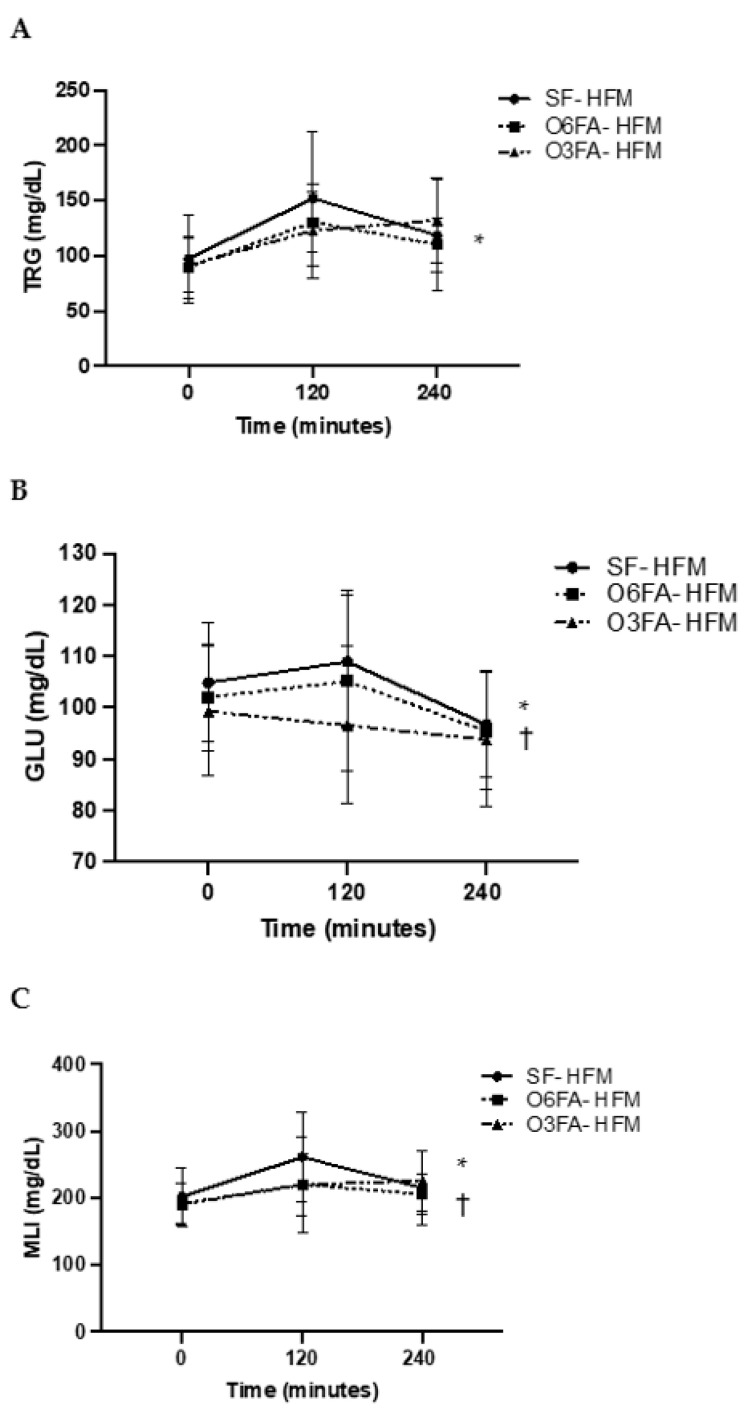
(**A**–**C**). Postprandial metabolic responses. Metabolic responses in the three smoothie conditions are displayed as a mean ± SE for baseline, 2 h (120 min), and 4 h (240 min). TRGs (**A**), GLU (**B**), and MLI (**C**) are displayed with the SF-HFM in shaded black circle, the O6FA-HFM in black squares, and the O3FA-HFM in black triangles. * Indicates significance across time points, and † indicates significance as an interaction between smoothie type and time point.

Postprandial TRGs, GLU, and MLI can be found on Figure 1A–C. There was a significant increase in TRGs across time points from the baseline to 4 h (*p* < 0.001). There was a trend toward a significant interaction between the TRG and SF and the O3FA and O6FA smoothies (*p* = 0.06), which was due to the increase in the SF-HFM compared to the O3FA smoothie at 2 h (*p* = 0.10); however, these findings did not reach significance. There was a significant increase in GLU across time points in all conditions (*p* = 0.002) and a significant effect by smoothie type (*p* = 0.036). Specifically, the SF smoothie had a significantly higher GLU response compared to the O3FA smoothie at 2 h (*p* = 0.034), even though they were matched for sugar content. There were no differences between the SF and O6FA smoothies or the O6FA and the O3FA smoothies. Due to the trend toward significance in TRGs and the higher postprandial GLU in the SF smoothie, there was a higher MLI as an interaction between time and condition (*p* = 0.05). This was driven by the greater MLI postprandially at 2 h in the SF condition compared to the O3FA condition (*p* = 0.041).

**Figure 2:**
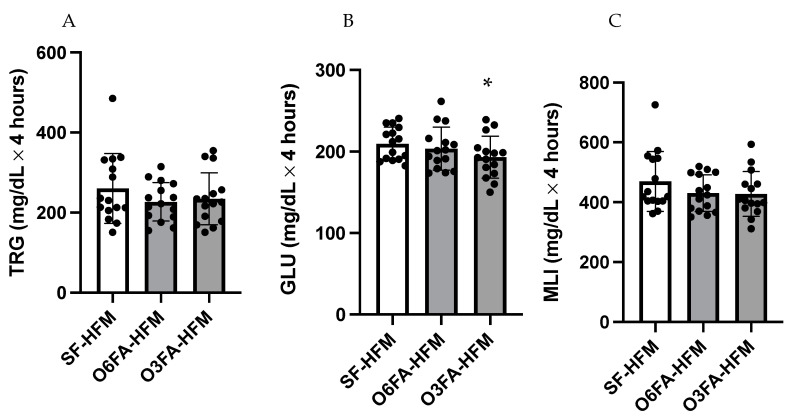
(**A**–**C**). Area under the curve for the postprandial metabolic responses. TRGs (**A**), GLU (**B**), and MLI (**C**) are displayed with each black filled-in circle representing individual participant data. * Indicates significance as an interaction between time and smoothie type.

AUC data are shown in Figure 2A–C for TRGs, GLU, and MLI. There was no difference in AUC for TRGs between conditions (*p* = 0.212); however, there was a significant difference in postprandial GLU AUC (*p*= 0.025), driven by a greater total AUC in the SF smoothie compared to the O3FA smoothie (*p* = 0.023). There was a trend toward a higher MLI in the SF compared to the O3FA condition (*p* = 0.077).

**Figure 3:**
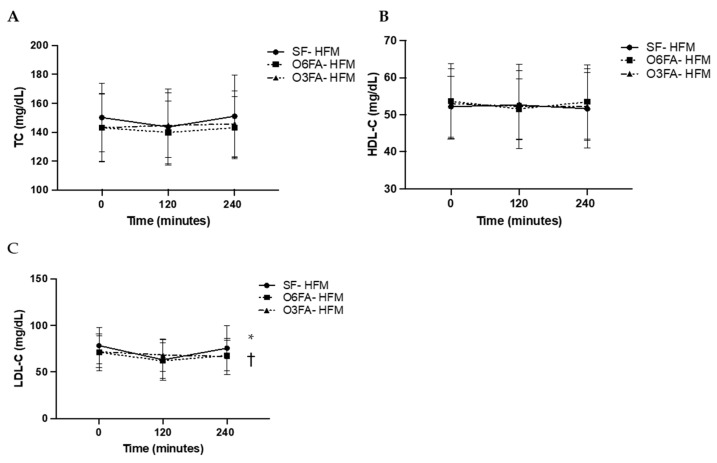
(**A**–**C**). Postprandial metabolic responses. Metabolic responses in the three smoothie conditions are displayed as a mean ± SE for baseline, 2 h (120 min), and 4 h (240 min). TC (**A**), HDL-C (**B**), and LDL-C (**C**) are displayed with the SF-HFM in shaded black circle, the O6FA-HFM in black squares, and the O3FA-HFM in black triangles. * Indicates significance across time points, and † indicates significance as an interaction between smoothie type and time point.

TC, HDL-C, and LDL-C are displayed is Figure 3A–C. There was no significant difference across time points for TC (*p* = 0.184), among smoothie types (*p* = 0.353), or as an interaction between time and smoothie type (*p* = 0.135). There was no significant difference across time points for HDL-C (*p* = 0.714), among smoothie types (*p*= 0.456), or as an interaction between time and smoothie type (*p* = 0.701). There was a significant difference across time points for LDL-C (*p* = 0.001). While there was no difference by smoothie type alone (*p* = 0.589), there was a significant interaction between LDL-C and smoothie type (*p* = 0.041). This was driven by the drop in LDL-C at 2 h (*p* = 0.013), followed by the increase from 2 h to 4 h in the SF condition (*p* = 0.046), which did not occur in the O6FA or O3FA conditions.

## 4. Discussion

### 4.1. Main Findings

The present study sought to determine whether there would be differential responses in TRGs, GLU, and MLI among smoothies with varying fatty acid profiles. There was a lower GLU response in the O3FA smoothie compared to the SF smoothie, along with a trend toward higher TRGs in the SF smoothie, resulting in a higher MLI following the SF smoothie. This is in support of our hypothesis that the metabolic load from the SF smoothie would be elevated compared to the other smoothie conditions. This indicated that not only is the total level of macronutrients important in determining postprandial responses, but also the subset of fat types. Because the postprandial lipemic response is an independent risk factor of CVD development, determining the impact of varying levels of dietary fatty acids in a true-to-life standardized meal is of critical importance.

### 4.2. Postprandial Responses among Smoothie Types

There have been many studies that have examined the impact of fat on postprandial TRGs, which have found that a response greater than 220 mg/dL is a clinical threshold in which myocardial infarction and CVD risk are increased [6,20,21]. The TRG response in participants in the present study was lower than this clinically established threshold. Because our participants were generally metabolically healthy, this may have limited our ability to detect differences across conditions due to the moderate increase in postprandial lipids. The participants in the present study did have similar levels of fasting TRGs and peak TRGs compared to similarly aged participants that we have previously assessed [11,22]. Young, healthy adults who are chronically active and have healthy fasting TRGs have higher lipoprotein lipase activity compared to other populations (i.e., older adults) [23] and therefore may not be as impacted by dietary fats in the postprandial period. With healthy liver function, they also likely to not have increased liver fat content that may be seen in other demographics [24], which also increases postprandial TRGs. These mechanisms could partially explain the low overall TRG response observed from young adults in the present study. In addition, the SF smoothie was made with butter and heavy cream. It is possible that there are different TRG responses and, consequently, a CVD risk when comparing dairy fat to meat SF [25]. Larger quantities of SF from meat sources may have exacerbated the postprandial lipemic response compared to the smoothie used in the present study.

Additionally, the postprandial responses in TC, HDL-C, and LDL-C were interesting, though not surprising considering the previous literature. There are conflicting results for LDL-C and HDL-C in the current literature, likely because these lipoproteins are composed of many subclasses [26] and there are differences in how they are reported in the existing literature. In our previous postprandial studies with nondiabetic, relatively healthy adults, TC seldomly changes postprandially [27]. HDL-C may increase postprandially [28], decrease [29], or not change across time points, likely due to the baseline triglyceride status, sex, and measured particle size [30]. However, even with minimal changes in the HDL particle number, previous research indicates that LDL-C may still decrease or increase postprandially [30]. The postprandial increase is likely due to increases in large LDL and VLDL particles and chylomicrons. Because smaller LDL particles are more atherogenic [31], it is difficult to say whether the changes in the calculated LDL observed in the SF condition from 2 to 4 h for this young and healthy population have clinical significance. In addition, in the present study, we were not able to measure the total number of particles or particle sizes. Because there are many subclasses of lipoproteins with various apolipoproteins on their surfaces, proposed mechanisms of action are purely speculative.

### 4.3. The Importance of Time Course in Evaluating Postprandial Outcomes

It is documented that when comparing SF to MUFAs, SF meals have a prolonged TRG response, and therefore we may have limited our ability to detect differences across fatty acid conditions due to the length of our postprandial period [16,32]. However, we also wanted to keep this work true to clinical assessments because 4 h meal challenges are commonly performed in medical practice. From the existing literature, it appears that MUFAs have an earlier peak response compared to SF [32]. While O3FAs and O6FAs are PUFAs, they did not have an earlier elevation compared to the SF smoothie used in the present study. While the evidence is unclear regarding TRG levels within the 4 h postprandial period window, given the time course of GLU metabolism postprandially, we should be able to see differences in the metabolic load within this timeframe, especially if the GLU measurements can be taken more frequently.

In a thorough review paper written by Monfort-Pires and colleagues, researchers suggest that differences between PUFAs and SF may be seen using a 6 to 8 h postprandial period [33]. They specifically state that using a 4 h postprandial period may limit the ability to detect differences in TRGs, though they do mention a trend toward an increase in TRGs within 4 h (*p* = 0.10). Their findings are consistent with the present study; however, we wanted to expand on these results and add in a GLU analysis. Because 4 h oral fat tolerance tests are commonly used in clinical practice [34], these results may have some clinical utility. If using a fat tolerance test to examine TRGs alone, a longer postprandial period may need to be used. However, if conducting research or clinical work utilizing a true-to-life standardized mixed meal, it is possible that differences can be observed when considering the metabolic load of that test meal. Yet the MLI across a 4 h period should be further investigated to assess the clinical utility because the GLU may be back at or near preprandial levels at 3 or 4 h after meal consumption depending on TRG and GLU clearance (discussed below). Finally, we would be remiss not to note that some studies have also found an increase in TRGs greater in MUFAs compared to PUFAs and SF [35]. Therefore, it is possible that TRGs are not as responsive to SF in young adults compared to a postprandial TRG assessment in other populations, including individuals with metabolic syndrome, obesity, or older adults [16,17,36]. Nevertheless, there was a trend toward an increase in the SF condition, and because we did not have a MUFA comparison, it is possible that it would have increased even more than the O3FA condition, though this is purely speculative without the direct comparison.

### 4.4. Postprandial Responses and Mechanisms of Action

A recent review paper examining the impact of O3FAs on CVD risk factors elucidates glucose mechanisms of action in various types of individuals. The authors highlight, as described above, studies that show no differences in postprandial glycemia, fasting plasma glucose, and glycated hemoglobin [37], while others show possible positive effects from O3FA dietary interventions on reducing the risk of diabetes development [38]. Excessive intake of FAs, particularly from SF, can impair insulin release and lead to B-cell dysfunction, ultimately increasing postprandial glucose. Because the SF smoothie contained 61% saturated fat, we believe that these mechanisms could lead to an acute increase in glucose output and a reduction in insulin clearance from the liver in the SF compared to the O3FA smoothie. In addition, if there is increased lipid uptake and TRG storage in the muscle due to the competing TRG and GLU clearance, there could be lower glucose oxidation [39]. In addition, in a recent study examining the effects of utilizing a high-omega-3 diet in mice, researchers reported significant reductions in postprandial GLU compared to diets with varying macronutrients. The authors state that the high O3FA diet may suppress blood glucose concentrations through reductions in gluconeogenesis [40]. While we were not able to detect precise mechanisms for glucose-lowering action in the current study, future research should elucidate specific mechanisms of action to evaluate the MLI from these FA test meals. Understanding the TRG and GLU responses is imperative considering individuals are consuming mixed meals every few hours throughout the day with snacks in between [9].

#### Experimental Considerations

GLU was taken in the present study at baseline, 2 h, and 4 h postprandially. Ideally, measurements would have been taken more frequently, including a 30-min and 1 h GLU assessment. However, this was part of a larger study investigating airway inflammation after various meal challenges, with outcome measures necessary to take in a 2 h time course. Additionally, we wanted to assess the utility of using MLI, which may translate to a mixed meal challenge that could be feasible to perform in a clinical setting. While future research should certainly examine the magnitude of the GLU response after meals of varying fatty acid compositions, this time course still provides vital information about postprandial glycemia, lipemia, and overall metabolic load.

## 5. Conclusions

A high O3FA smoothie lowers postprandial GLU and may lower postprandial TRGs compared to a SF smoothie, resulting in an increased MLI associated with the standardized SF smoothie. It is important to better understand how to apply these results clinically, considering the 4 h postprandial period is commonly used, and to consider incorporating mixed meal challenges high in fat to assess lipemia, glycemia, and MLI. In addition, it is vital to elucidate the mechanistic underpinnings in the differences in postprandial responses among FA types to better design therapeutic interventions targeting TRG and GLU metabolism.

## Figures and Tables

**Table 1:** Nutritional information for each HFM smoothie.

	O3FA-HFM	O6FA-HFM	SF-HFM
Kcal (g/kg)	12.01	12.02	12.10
Total fat (g/kg)	0.83	0.83	0.82
Sat fat (g/kg)	0.11	0.11	0.51
Omega-6 (g/kg)	0.06	0.56	0.02
Omega-3 (g/kg)	0.40	0.00	0.01
Total carbohydrate (g/kg)	1.07	1.07	1.06
Sugar (g/kg)	1.02	0.98	1.02
Protein (g/kg)	0.07	0.07	0.07
Sat Fat (% of Total Fat)	14.07	12.50	61.28

**Table 2:** Participant characteristics.

	*n =* 15 *(*M* = *6*, *F* = *9*)*
	Mean ± SD
Age (years)	21.9 ± 1.5
Height (in)	67.9 ± 3.7
Weight (kg)	74.7 ± 14
Waist Circumference (cm)	78.6 ± 10.4
Body Mass Index (kg/m^2^)	25.3 ± 5.6
Body Fat (%)	25.8 ± 10.8
Time in MVPA (hours/week)	6.6 ± 2.7
